# Fermenting ideas at the interface of wine yeast biotechnology and synthetic genomics

**DOI:** 10.1093/femsyr/foag006

**Published:** 2026-01-27

**Authors:** Isak S Pretorius

**Affiliations:** The Chancellery and ARC Centre of Excellence in Synthetic Biology, Macquarie University, Sydney, NSW 2109, Australia

**Keywords:** wine yeast biotechnology, synthetic yeast genomics, Yeast 2.0, retrospective

## Abstract

Looking back over nearly five decades in yeast research, I often marvel, over a glass of wine, at how a single-celled organism like yeast has taken me on such a remarkable journey. We have travelled across four continents, using basic science and applied biotechnology, and into the heart of international collaborations that continue to redefine what is possible in biology. When I was growing up on a farm in rural South Africa, I never imagined that yeast would become both my lifelong research companion and my passport to the world of scientific discovery and innovation. Yeast taught me that impactful research should be directed toward increasing fundamental understanding in a context responsive to the applied needs of end-users, at both the level of problem selection and experimental design. Writing this *Retrospective* is therefore both an honour and an opportunity to reflect not only on the science and where it is leading but also on the people, places, and serendipitous moments that have shaped my career.

## From humble beginnings to the frontiers of scientific discovery

### Inspired by heritage, legacy and a pioneering spirit

In 1666, Johannes Pretorius—a young man from Ouddorp in the Netherlands, born on 26 October 1642 to a German shoemaker, Barend Wesselszoon Schulte, and his Dutch wife, Aaltje Jansdochter—arrived at Kaap de Goede Hoop (the Cape of Good Hope), becoming one of the earliest Dutch settlers in southern Africa. From these humble beginnings, the Pretorius family tree grew in influence and prominence, producing leaders, pioneers, and visionaries whose name became inseparably linked to South African history; so much so that the capital city Pretoria (founded in 1855 in the then Boer republic known as the Zuid-Afrikaansche Republiek, ZAR) was named in honour of Andries Pretorius a legendary *Voortrekker* leader (and fifth-generation descendant of Johannes Pretorius).

#### A time of tragedy and deep sacrifice

**Figure 1:**
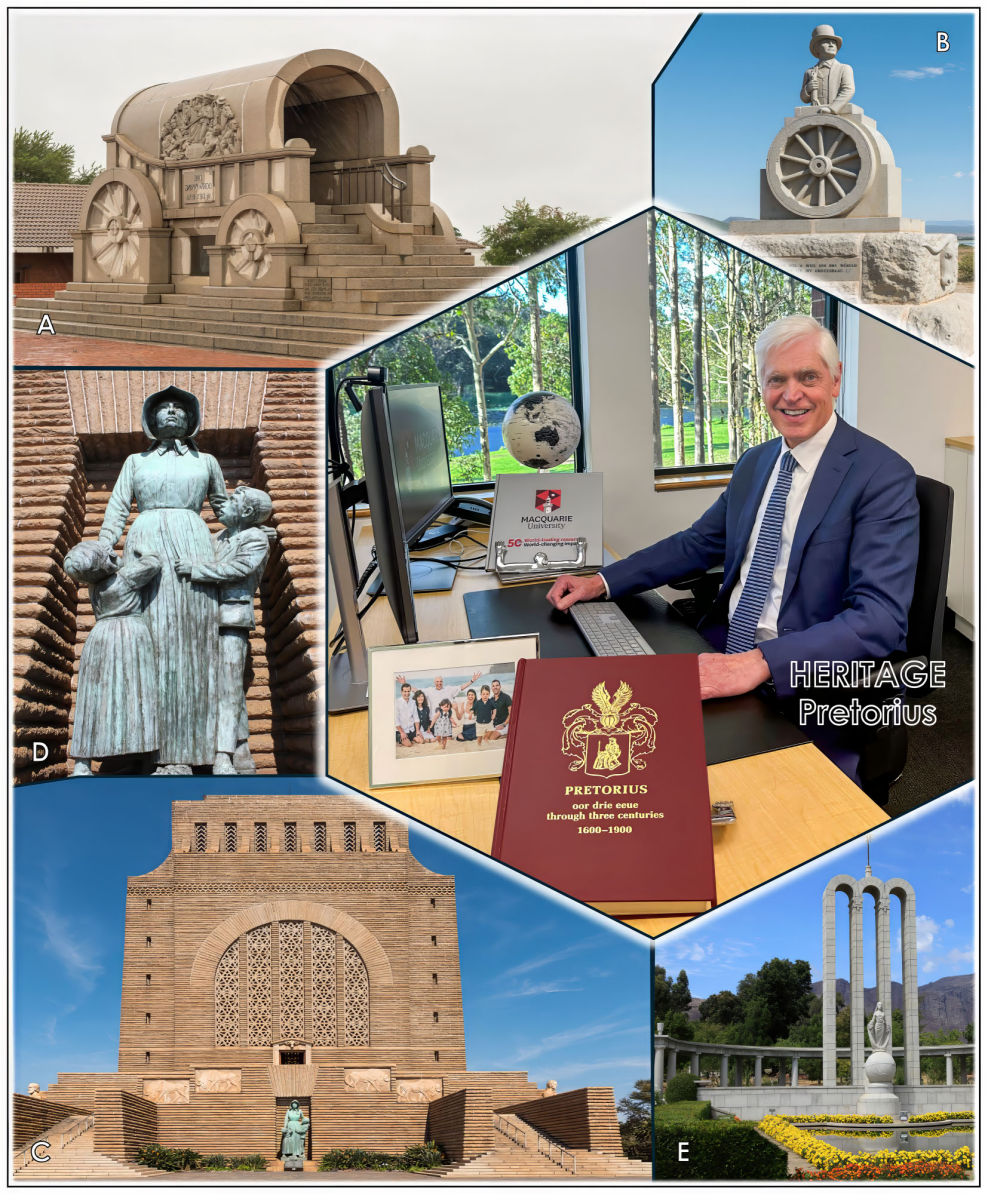
The Pretorius family heritage spans more than 350 years in South Africa. A fifth-generation descendant of the founding ancestor, Johannes Pretorius—who arrived at the Cape of Good Hope in 1666—the *Voortrekker* leader Andries Pretorius played a defining role in shaping the destiny of the Afrikaner people, themselves descended from Dutch, German, and French Huguenot settlers of 1652–1688. The figure shows significant historical monuments: (A) an ox wagon memorial commemorating a decisive victory led by Andries Pretorius on 16 December 1838 over Zulu Chief Dingane at the *Battle of Bloedrivier* in Natal; (B) a statue of *Andries Pretorius*, after whom the capital city Pretoria was named in 1855; (C) the *Voortrekker Monument* in Pretoria commemorating the 1838 Great Trek; (D) the Women and Children memorial in Bloemfontein as a remembrance of some 26 000 women and children who died in British concentration camps during the second Anglo-Boer War (1899–1902); and (E) a memorial in Franschhoek commemorating the 1688 French Huguenots.

Andries Pretorius shaped the destiny of the Afrikaner people, leading the Great Trek from the Cape northward into the Orange Free State (OFS), Natal and the Transvaal securing a decisive victory at the Battle of Bloedrivier (‘Blood River’) in 1883. For many Afrikaners, Andries Pretorius symbolised vision, courage, resilience, faith, a deep sense of destiny, and the struggle for self-determination (Fig. 1). After his death in 1853, his son, Marthinus Wessel Pretorius, became the first President of the ZAR (first from 1857 to 1860, and again from 1864 to 1871). For a brief period (1859–1860), MW Pretorius held the presidency of both the ZAR and the OFS, attempting to unite the two Boer republics under a single leadership. This dual role proved politically challenging, and he eventually resigned from the OFS presidency to focus on the ZAR (Transvaal).

During the Second Anglo-Boer War (1899–1902), some 26 000 Afrikaner women and children died of starvation and disease (Fig. 1); a tragedy that nearly broke the spirit of those early pioneers. My great-grandparents fought in this war, and their children—my grandparents—were fortunate to survive and be able to rebuild their lives on the ruins of a scorched land.

#### Forming a new nation

Following the Anglo-Boer War, life was difficult in the newly established Union of South Africa (1910) and through World War I (1914–1918). My father, Barend Johannes (Ben) Pretorius, and mother, Hannetjie (neé Du Plessis, a surname connected to 200 French Huguenots who fled the severe consequences of the Edict of Nantes in Europe and settled in the Cape in 1688; Fig. 1), were born in the mid-1920s and grew up during the Great Depression (1929–late 1930s) and World War II (1939–1945). In 1948, Afrikaners gained political power, and in 1961, South Africa formally became the Republic of South Africa (RSA), gaining independence from Britain.

Nearly four centuries after the first Pretorius settler arrived in the Cape, I (as a 12th generation Pretorius) take inspiration from the pioneering legacy and spirit of my forebears and apply it in a very different arena—not governance or conquest, but education and science.

### Grounded in the land, lifted by learning, and tested by service

#### Education above all

**Figure 2:**
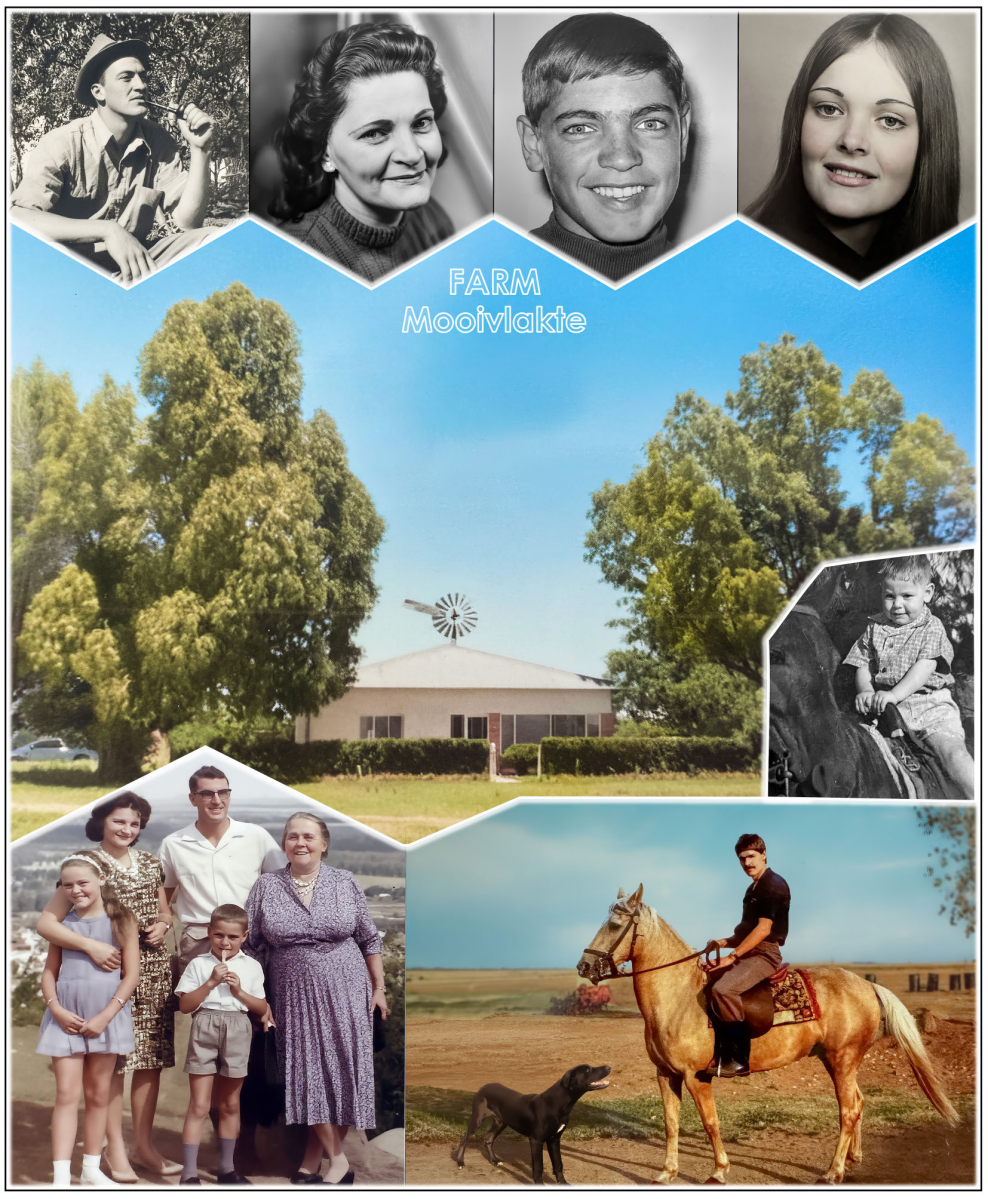
Growing up on *Mooivlakte*, a small subsistence farm in rural South Africa, I was raised in a supportive and loving family. Top row (left to right): my father, Ben; my mother, Hannetjie; me (Sakkie); and my sister, Heidi. Bottom left: the Pretorius family, including our live-in grandmother, Bettie. As a toddler I learned to ride my black pony, *Sieraad*. Later, my palomino horse, *Vrystaat*, and our dog, *Fronsie*, became my inseparable companions on the farm in the province of the OFS.

**Figure 3:**
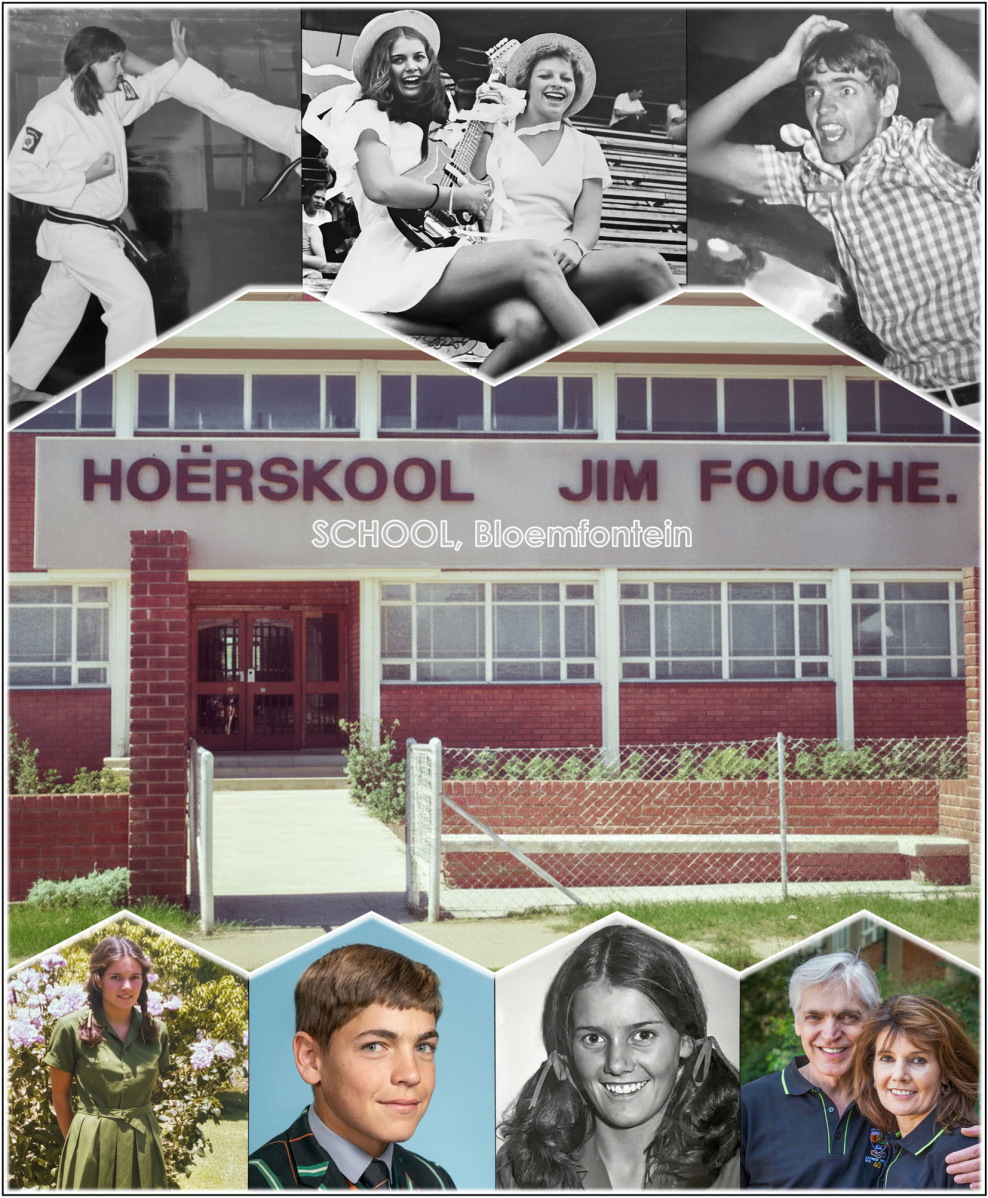
Formative school years at Jim Fouché High School in Bloemfontein where I met my future wife, Elize. Top row (left to right): Elize earning her black belt in karate; as vice-school-captain and cheerleader (1977); and Sakkie as a school-captain and cheerleader. Bottom row (left to right): Elize; Sakkie; Elize; and the two of us together at Sakkie’s 40th School Reunion.

Despite the economic hardships my subsistence-farming parents endured (including never owning their own property), they placed the education of their children—my sister Heidi and me—above all else (Fig. 2). Thanks to their values-based parenting, and the support of my grandmother, Bettie Pretorius (née De Klerk), and my schoolteachers, I was dux scholar during my primary and secondary school years in and around Bloemfontein, the capital of the province of the OFS. I was young when my parents, grandmother and teachers instilled in me a deep desire to live a life I’d be proud to share. Integrity and excellence became my DNA’s double helix. At school, I took part in athletics and rugby. In Year Twelve (1975), I played wing for my school’s First Rugby Team and was elected School Captain of Jim Fouché High School. At school, when I was age 16, I met 13-year-old Elize Olivier: the woman I would eventually marry. With the gift of hindsight, I recognize as a rare enduring privilege having found my life partner so early in life (Fig. 3).

#### It was the letters that sustained me

**Figure 4:**
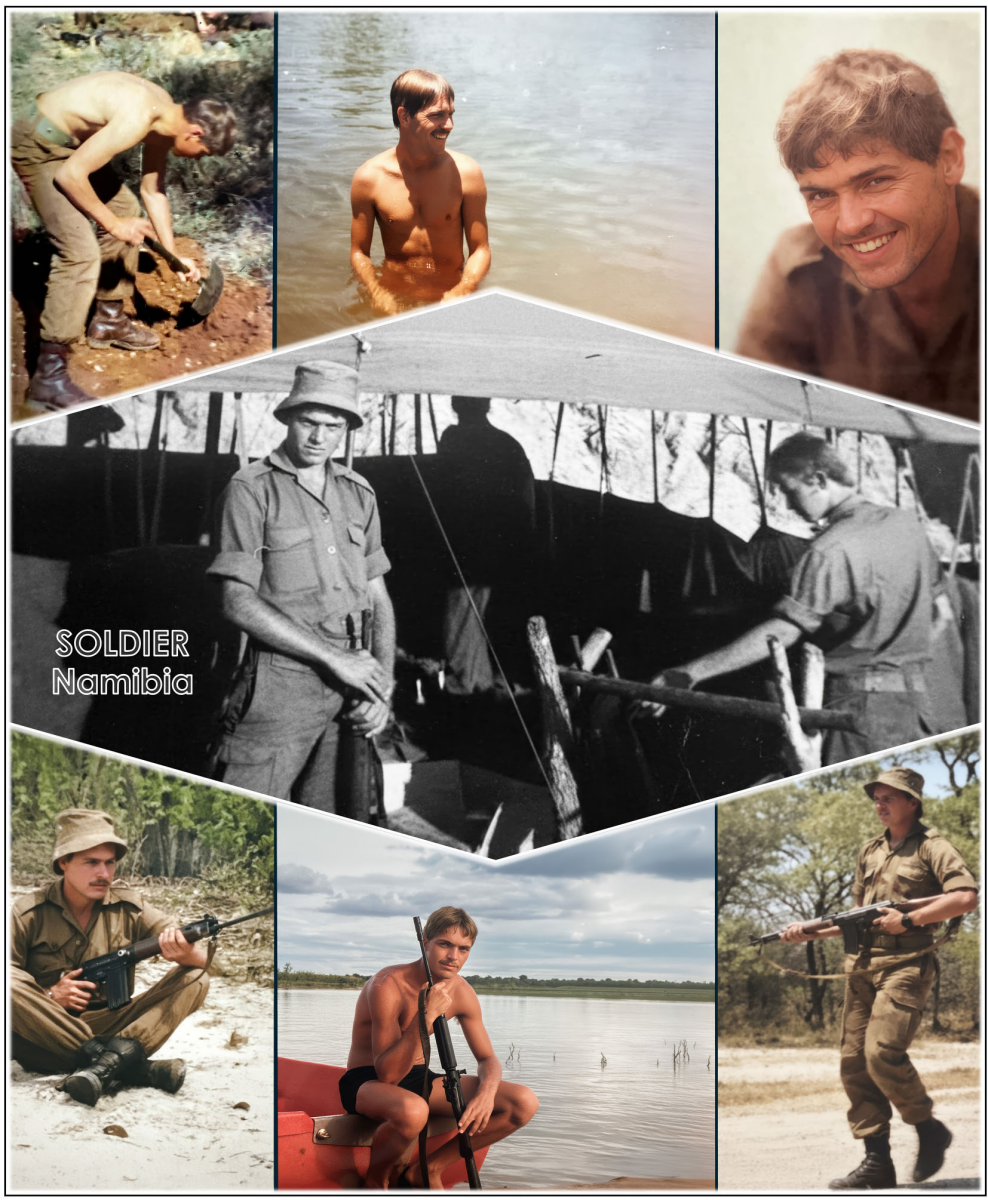
Conscripted into the South African Defence Force and deployed to the Namibia–Angola border. In 1976, I served as Rifleman 1 in Platoon 1 of the First South African Infantry Battalion (1 SAI) near Eenhana. Over the following eight years, during my annual reserve call-ups while studying at university, I was posted to several locations, including Bagani in Namibia’s scenic Caprivi Strip—a narrow corridor bordered by Angola, Zambia, Zimbabwe, and Botswana.

In 1976, I was conscripted into the South African Defence Force, serving as Rifleman 1, Section 1, Platoon 1, C Company of the 1st South African Infantry (1 SAI) Battalion in Bloemfontein. My training prepared me, among other skills, to be a sniper, a role demanding patience, precision, and physical resilience. Within nine weeks of basic training, I was deployed to an army base camp near Eenhana, a small village on the northern border of South-West Africa (now Namibia) (Fig. 4).

This deployment came at the tail-end of the Angolan Revolution (gaining independence from Portugal) and amid the Cold War. Across the Namibian border, a Soviet Union proxy army of some 55 000 Cuban soldiers supported the MPLA (People’s Movement for the Liberation of Angola), while China backed the FNLA (National Front for the Liberation of Angola), and Western powers supported UNITA (National Union for the Total Independence of Angola). My task as a sharpshooter was to infiltrate enemy lines and neutralize key communication systems; a role that required me to remain calm under pressure, move silently, and act decisively. Each successful mission tested not only my skill but also my nerve and endurance.

What carried me through those tense times were the letters from Elize, sent daily and sometimes accumulated in bundles of thirty, which I eagerly read whenever our platoon returned to base for brief respites. The camaraderie among my fellow soldiers, along with the stunning African bush, provided moments of relief from the emotionally challenging, unrelenting pressure of active service.

#### War and a fish-eagle teaching me life skills

During my reserve call-ups over the next eight years while I was a university student, I served in locations such as Bagani in Namibia’s Caprivi Strip, a region bordered by Angola, Zambia, Zimbabwe, and Botswana. There, I observed—and came to admire—the African fish-eagle (*Haliaeetus vocifer*), a bird that soars boldly into storms while other birds and smaller animals seek shelter. From high above the tempest, the fish-eagle spots its prey with unerring accuracy. That image has stayed with me: a lesson in strategic focus, perspective and total commitment, teaching that even in turbulent times, clarity of vision and precision can lead to success.

**Figure 5:**
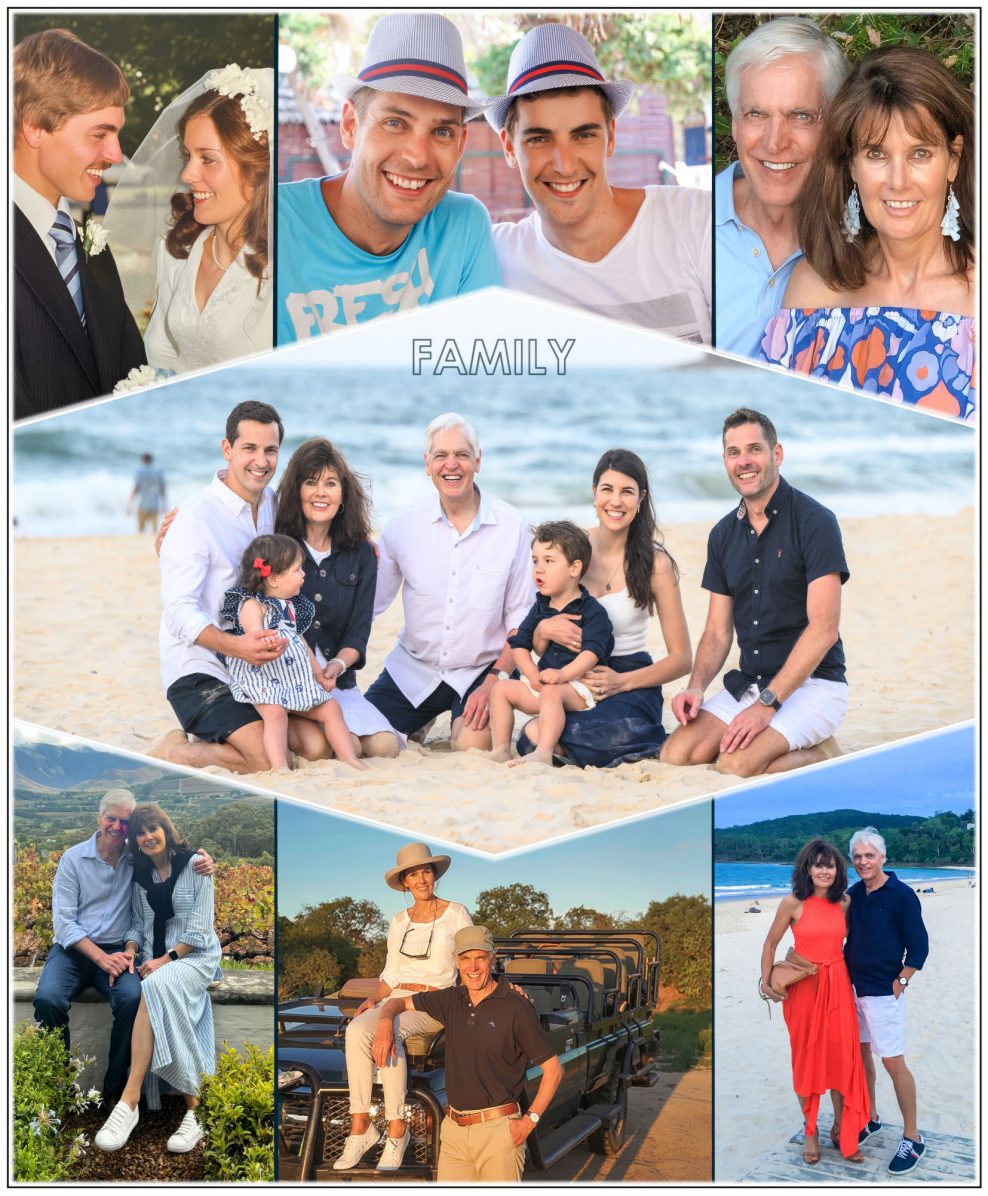
The closely-bonded Pretorius family. Top row (left to right): Elize and Sakkie’s wedding; our two sons—Bernard (born in 1979 in Bloemfontein) and Stefan (born in 1988 in Stellenbosch); and Elize and Sakkie in later years in Australia. Middle panel: our family with Stefan’s wife, Stephanie, and our Sydney-born grandchildren, Louis (born in 2021) and Giorgia (2022). Bottom row (left to right): three of our ‘happy places’: the Boland in the Cape winelands; the Sabi Sands Game Reserve in the Bushveld near to the Kruger National Park; and Noosa Heads on the Sunshine Coast in Queensland.

Those years of military service, though challenging and sometimes harrowing, shaped me profoundly. They instilled in me a deep sense of purpose, discipline, respect, integrity, loyalty, resilience, and a calm focus under pressure; qualities that would later prove invaluable in the laboratory and in leadership. The long nights in the bush demanded patience and vigilance, just as scientific discovery requires meticulous observation and perseverance. The camaraderie of the troops taught me the power and collective edge of genuine collaborative teamwork, while the fish-eagle’s bold flight into the eye of a storm and its devotion to its young became a lifelong metaphor for embracing challenges with courage and clarity. These principles have been guiding tenets in my approach to family: with my love and commitment to my wife Elize and in parenting our two sons, Barend Johannes (Bernard; born in 1979) and Isak Stefan (Stefan born in 1988). We are a close-knit family and a formidable unit when faced with any challenge life can throw at us (Fig. 5).

#### From rifle scope to microscope—a new wonderful journey

After the fall of the Berlin Wall in 1989, and the crumbling of communism and the Soviet Union, a rare opportunity emerged from South Africa’s political system to change course. Early 1990, President FW de Klerk released Nelson Mandela as a political prisoner—who then became South Africa’s President in 1994. During his presidency, the Rainbow Nation was born, after he made a legendary appearance when the Springboks won the Rugby World Cup against New Zealand’s formidable All Blacks team in 1995. Tears of joy swept across the country.

When the Cold War and my time in uniform ended, I carried with me not only the relief of survival but also a sharpened sense of purpose, and a deeper appreciation of nature and my family. I was ready to redirect my energy into understanding the mysteries of life. My fascination with farm animals (especially horses) and wildlife ultimately led me to an interest in biology at its smallest scales. Where once I had peered through the scope of a rifle, I now focussed my gaze through the lens of a microscope, seeking answers hidden in the genetic code of yeast. This shift marked the beginning of a new journey; one no less demanding than the military, but infinitely more creative, inspiring and world-shaping: the pursuit of science. Just as my forebears carved their path in a new land, I have sought to explore uncharted territories in microbiology, discovering, harnessing, and shaping the microscopic worlds of yeast that, in their own way, influence human life across the globe.

### Moulded by study, hard work, and servanthood leadership

#### A taste for the cutting edge

**Figure 6:**
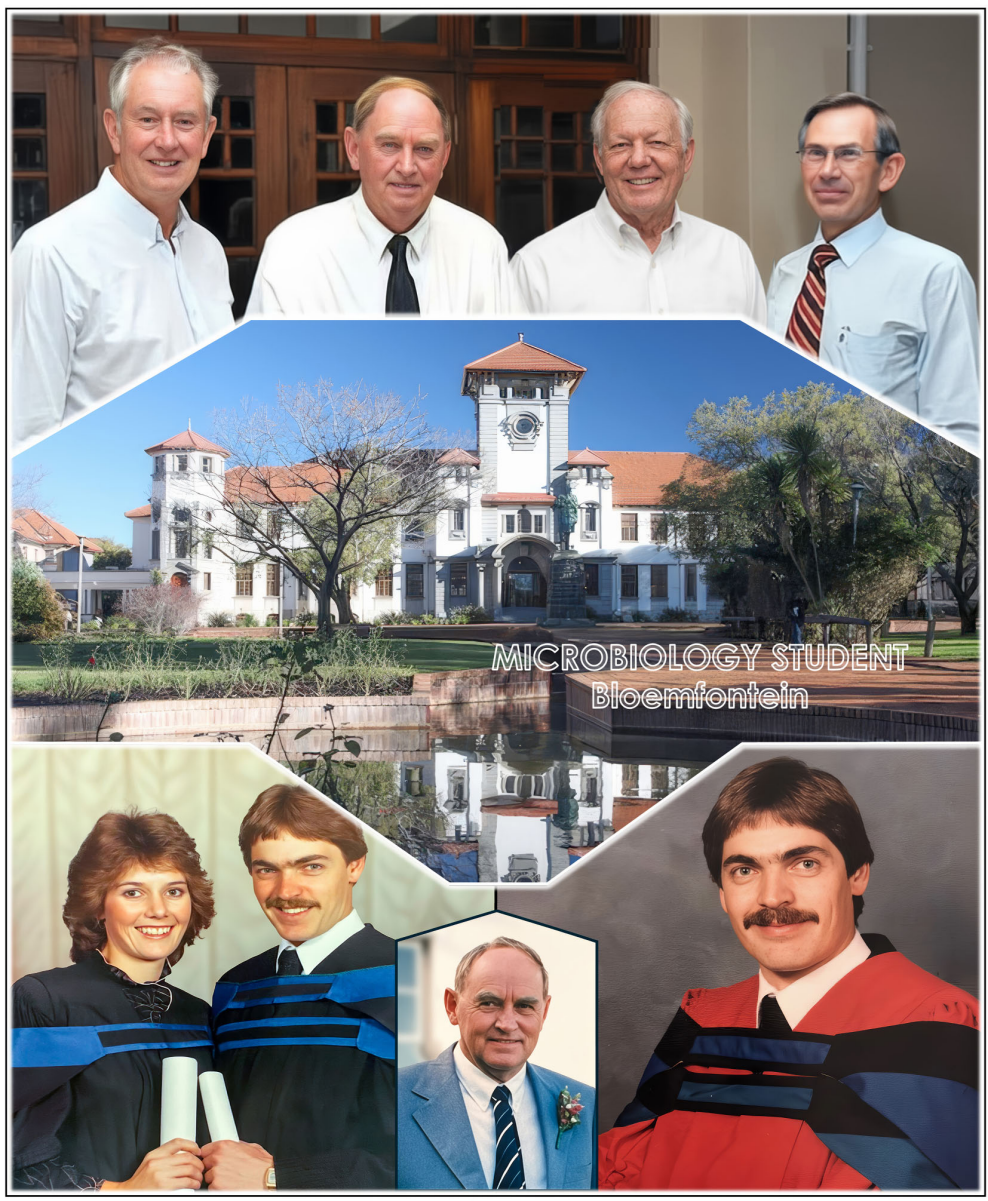
Student years at the UOFS in Bloemfontein (1977–1986). Top row (left to right): Elize and Sakkie’s microbiology professors—Bernard Prior, Piet Lategan, Hennie van Vuuren, and James du Preez. Bottom row (left to right): Elize and Sakkie graduating together in microbiology at the UOFS, the cradle of yeast-fermentation education in South Africa; Professor Piet Lategan, the visionary Head of the Department of Microbiology; and Sakkie completing his doctoral degree (1986) jointly undertaken at the UOFS and the Albert Einstein College of Medicine in New York.

**Figure 7:**
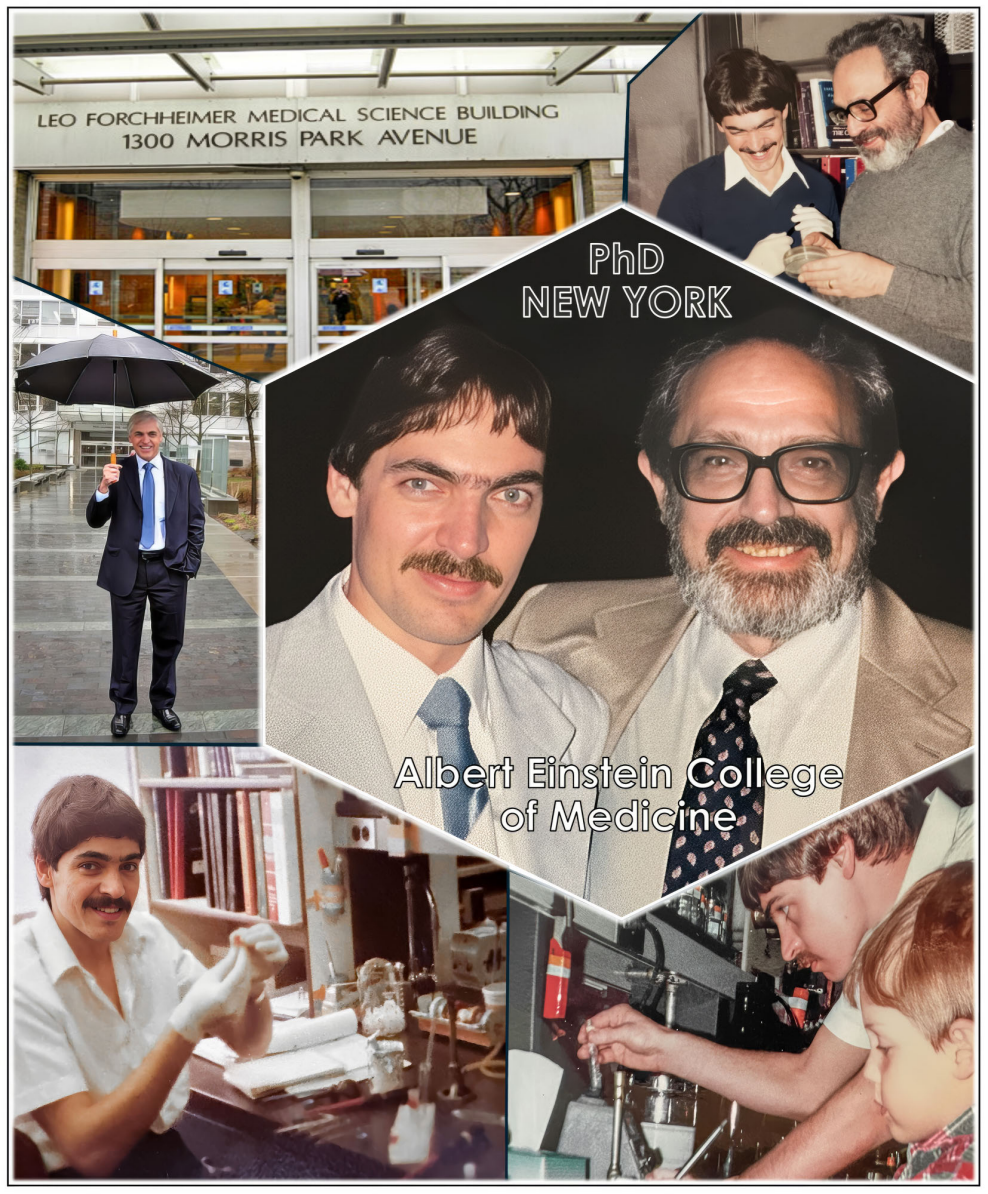
Transformative years as a doctoral student and researcher at the Albert Einstein College of Medicine in New York. Sakkie had the privilege of learning and conducting research under Professor Julius Marmur, the world-renowned molecular geneticist who, in 1961, became the first to isolate DNA, separate the two strands of the double helix, and successfully reanneal them—pioneering work that paved the way for the deciphering of the genetic code by Nirenberg, Matthaei, and Khorana in 1966.

At the University of the Orange Free State (UOFS), I pursued tertiary education and achieved the BSc.Agric (1977–1980), Hons.BSc. Agric (1981), and MSc.Agric (1982–1983) degrees with distinction (*cum laude*)—each growing my fascination for microbiology (Fig. 6). My microbiology professors—Piet Lategan, Bernard Prior, Hennie van Vuuren, James du Preez and Johannes van der Walt—inspired me to appreciate the wonder-worlds of yeast and fermentation. My PhD research, however, opened an entirely new horizon. With the significant support of wise mentors—Professors Piet Lategan in South Africa and Julius Marmur at the Albert Einstein College of Medicine (AECOM) in New York—I embarked on molecular yeast genetics at a time when this emerging field was expanding rapidly. Completing my PhD in 1986—split between Bloemfontein and New York—gave me not only a solid grounding in rigorous science but also a taste for cutting-edge research anchored in international collaboration that would shape the rest of my career (Fig. 7).

#### Establishing a research base in molecular yeast genetics in South Africa

##### Connecting curiosity with practical applications

**Figure 8:**
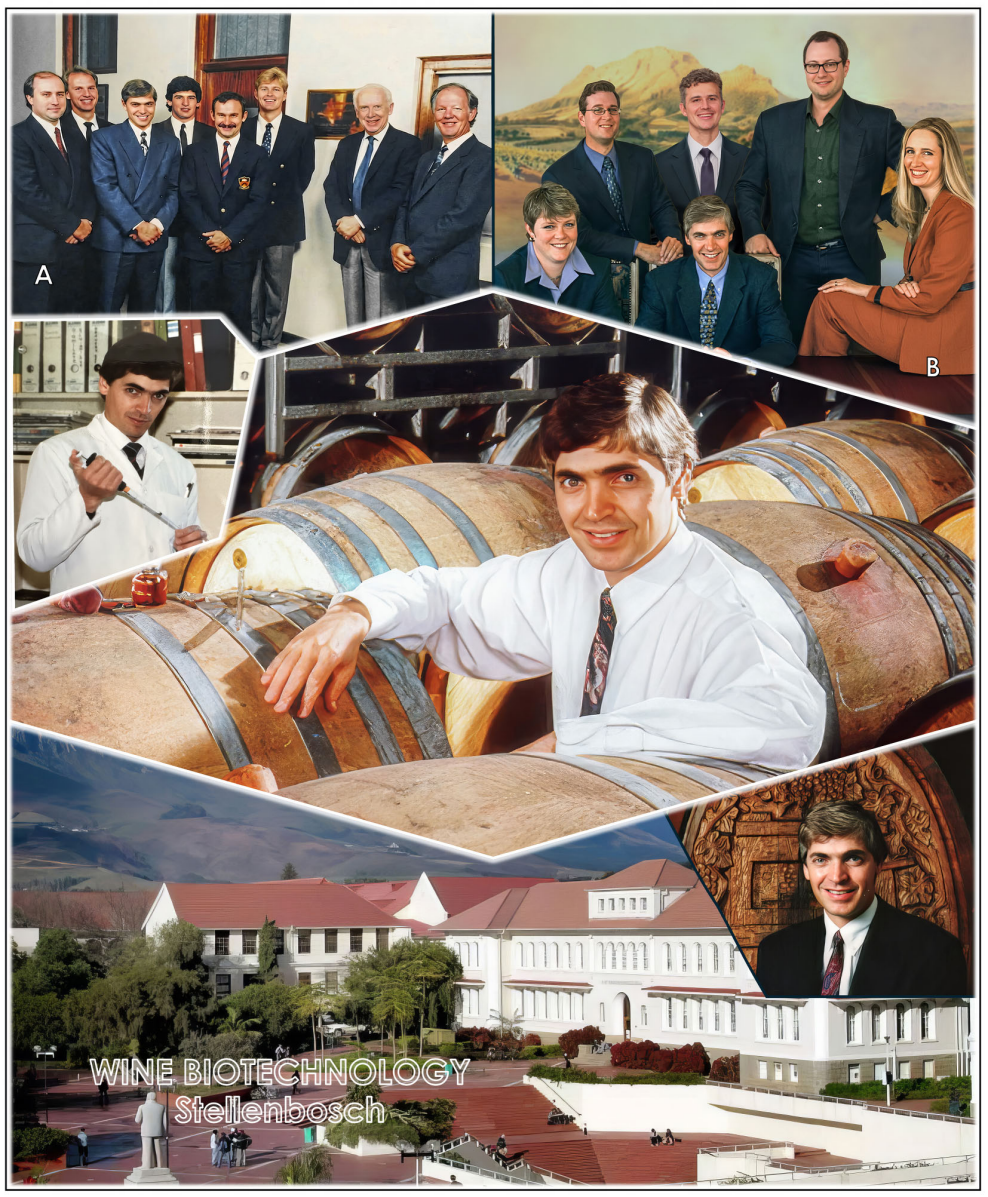
Successful years as a lecturer and young professor at Stellenbosch University (1987–2002). Top left (left to right): colleagues in the Department of Microbiology, Leon Dicks, Bernard Janse, Sakkie Pretorius, Adri Steyn, Emilé van Zyl, Marius Lambrechts, James Watson (who travelled from Cold Spring Harbor to open our new laboratories), and Hennie van Vuuren. Top right (left to right): Maret du Toit, Pierre van Rensburg, Marius Lambrechts, Florian Bauer, and Melané Vivier, who helped Sakkie establish the Institute for Wine Biotechnology (IWBT) at Stellenbosch University in 1995. Sakkie served as Professor of Wine Science and the founding Director of the IWBT, now known as the South African Grape and Wine Research Institute.

In 1987, I joined Stellenbosch University as a Senior Lecturer in Microbiology, and worked alongside a lifelong friend, Hennie van Vuuren, who fostered my entrepreneurial spirit. Those early years at Stellenbosch were formative (Fig. 8). My colleagues and students created a vibrant environment which allowed me to grow into an independent research leader, and eventually a full professor (1993). We explored polysaccharide-degrading *Saccharomyces cerevisiae* yeast strains, initially for bioethanol production and later for wine applications. My first postgraduate students and postdoctoral research fellows laid the foundations for a research program that connected curiosity-driven yeast genetics with practical applications in the bioethanol and fermented beverage industries.

During this period, I enjoyed valuable stints abroad, particularly at AECOM under a major NIH grant, and, in 1993, at the Max Planck Institute for Biophysical Chemistry in Göttingen with support from an Alexander von Humboldt Fellowship and the tremendous generosity of my host, Professor Dieter Gallwitz. Alongside my role as a professor at Stellenbosch University, I was also appointed as a part-time professor at the Katholieke Universiteit Leuven in Belgium (1995–2002). Here, I taught a course in Fermented Beverages, and co-supervised graduate students. These fruitful opportunities reinforced my belief in the value of international exchange and cross-pollination of ideas.

##### Better wine with better yeast

Back in Stellenbosch, the success of the patented industrial VIN13 wine yeast strain was a turning point. It demonstrated the power of yeast genetics combined with classical yeast strain development not only to advance knowledge but also to create tangible benefits for the global wine industry. Building on this momentum, I drove the establishment of South Africa’s first research base in molecular yeast genetics and biotechnology at Stellenbosch University. In 1995, I became the inaugural Director of the Institute for Wine Biotechnology at Stellenbosch University, where we pushed the frontiers of yeast strain improvement for the South African wine industry (see Pretorius 2000 and references therein). Our motto was that the best wine is yet to be made!

##### The first female director

During our years at Stellenbosch University, Elize advanced her studies and completed a doctoral degree in the molecular genetics of the plant-pathogenic bacterium *Xanthomonas chrysanthemi* (now *Pectobacterium chrysanthemi*) under the supervision of Martin Hattingh. She later became the Director of Stellenbosch University’s Research and Development Unit, followed by senior industry roles as Director of Innovation & Novel Product Development—first at Stellenbosch Farmers’ Winery and subsequently at Distell. In the corporate world, Elize refined her leadership skills and expertise, and was nominated as the first female board director of Distell, one of the world’s Top Ten wine companies. We both hold very fond memories of our years in Stellenbosch (1987–2002).

##### So hopeful

In the years immediately following President Mandela’s ascension to leadership, I was genuinely hopeful that the vision of a Rainbow Nation would continue to take root, thrive and mature. Like many South Africans, I wanted very much to believe that the social compact forged during that extraordinary transition would translate into a stable, inclusive, and secure future for all. However, as the late 1990s turned into the early 2000s, I became increasingly concerned about the direction in which our beloved country was moving. The social fabric began to fray, governance began to weaken, public institutions were showing signs of strain, and essential infrastructure and services started to falter. Personal safety and security became everyday concerns for many families. These were not abstract anxieties; they were lived realities that shaped daily life and long-term outlooks.

##### Not for us but our sons

As Elize and I became increasingly concerned, the decisive consideration was not about us but our two sons. We found ourselves asking not where we could continue to succeed and grow professionally, but where would our children have the best chance of growing up safely, freely, and with confidence in the future. We loved South Africa deeply—and still do—but we could no longer see a clear or prosperous path ahead for them to thrive as we had. Perhaps there was such a path, but search as we may, we could not see it.

##### Leaving with gratitude and a love that will never die

When the opportunity arose to move to Australia, it presented itself less as an escape and more as a responsibility: to act while we still could, rather than to wait until circumstances forced our hand. Leaving was painful, and we did so with gratitude rather than bitterness, carrying with us a profound respect for the people, mentors, and students who shaped our early careers. With the benefit of hindsight, we believe it was the right decision for our family, even though it meant closing a significant chapter of our lives. It was the hardest decision of our lives. We are still part of Africa, and Africa will always remain in us and our family here in Australia.

#### Exploring new horizons in Australia

##### High risk but too good to resist

**Figure 9:**
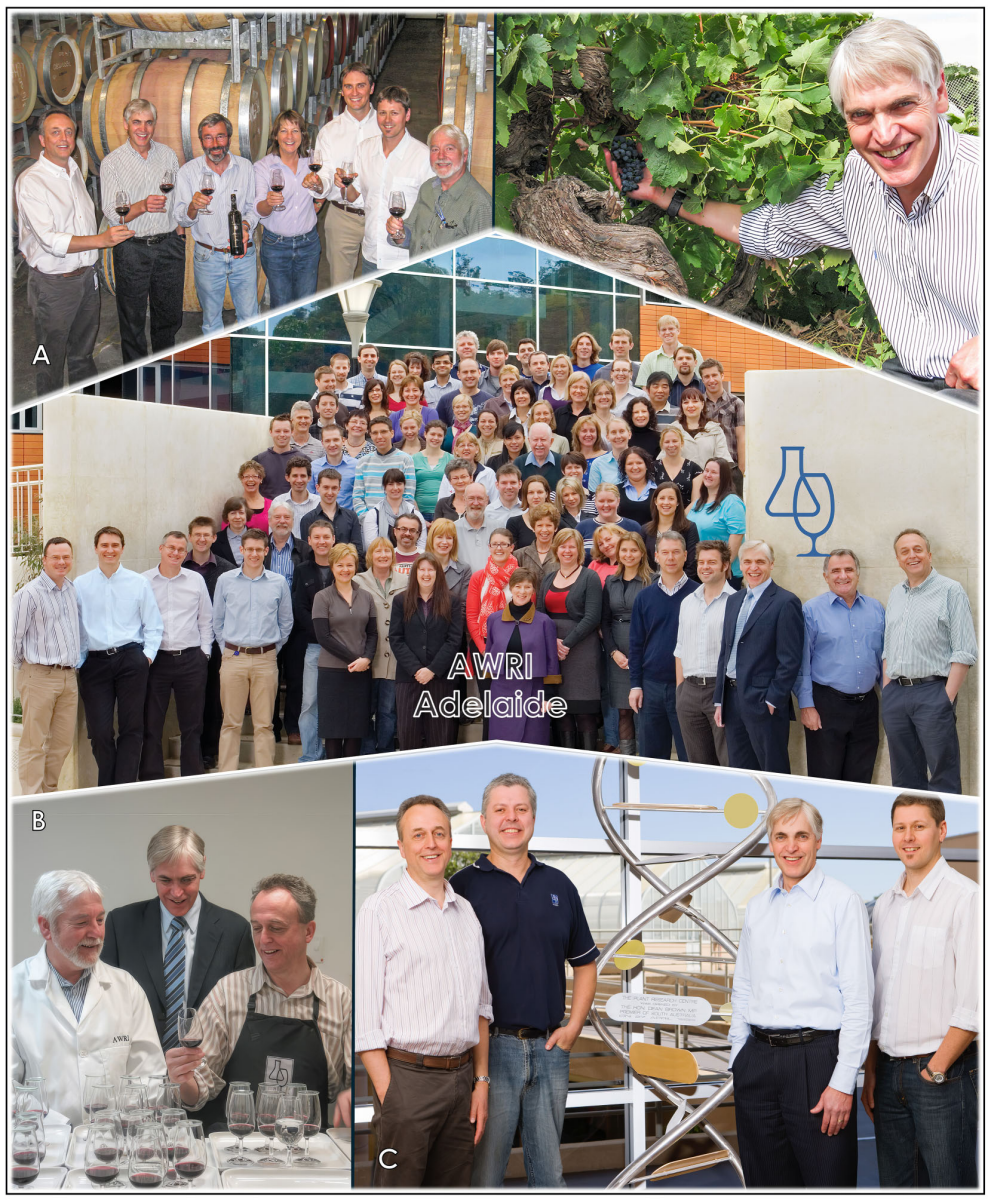
Leading the AWRI as Managing Director in Adelaide (2003–2011). (A) Visiting the Henschke winery and the historic ‘Grandpa’ vineyard, planted in 1886 in the Barossa’s Eden Valley (left to right): Paul Chambers, Sakkie Pretorius, Stephen Henschke (winemaker), Prue Henschke (viticulturist), Dan Johnson, Anthony Borneman, and Paul Henschke. (B) Analysing wines with Paul Henschke (left) and Paul Chambers (right). (C) The AWRI yeast team who sequenced the first genome of a wine yeast strain of *Saccharomyces cerevisiae* (left to right): Paul Chambers, Angus Forgan, Sakkie Pretorius, and Anthony Borneman.

In 2003, I moved with my family to Australia, and in 2004, accepted the role of Managing Director at the Australian Wine Research Institute (AWRI; a company limited by guarantee). Moving my family to Australia was a big and risky step, but the opportunity to succeed Peter Høj to lead a world-renowned wine R&D (research and development) company dedicated to the wine sector was irresistible (Fig. 9).

##### The Art of wine and the science of yeast

Since its inception in 1955, the AWRI has been isolating, selecting, and developing *Saccharomyces* and non-*Saccharomyces* yeast strains, as well as combinations thereof, to provide Australian winemakers with options to differentiate their wine styles while conducting risk-free fermentations. I was keen to build on the AWRI’s earlier pioneering work on nitrogen assimilation by wine yeast to modulate the undesirable production of hydrogen sulfide (H_2_S) in nitrogen-deficient grape-musts. I was also interested in expanding nongenetic modification (non-GM), genetic modification (GM), and molecular genetic approaches in the AWRI’s strain improvement program to identify yeasts with risk-free fermentation performance, delivering desired flavour attributes, or lowered alcohol concentrations (see Pretorius and Høj 2005 and references therein).

We used non-GM approaches (e.g. cross-breeding and mutagenesis) to generate novel and improved yeast strains by isolating dominant strains from freshly fermented wines with outstanding sensory characteristics. With our molecular biology approach, we gained a deeper understanding of which strains perform better under Australian winemaking conditions. Our AWRI team combined deep science with industry impact: sequencing the first complete genomes of an industrial *S. cerevisiae* wine yeast and of the most common wine spoilage yeast, *Brettanomyces bruxellensis*; engineering yeasts with enhanced aroma profiles; reduced ethanol levels in wine; and the ability to produce raspberry ketone in wine. While in the marketplace wine consumers continue to resist products resulting from genetic modification, experimental GM yeast research uncovered new knowledge and insights into the molecular intricacies of yeast cells. These experimental GM strains were used to validate certain concepts in the laboratory, thereby informing our non-GM strain development program. An example of how the two approaches came together is in the work undertaken with *Brettanomyces*. While one group of the AWRI team was investigating which *Brettanomyces* strains were the most prevalent and most resistant to winemakers adding sulfite (SO_2_), our molecular biology team was investigating ways to increase the addition of SO_2_ to kill *Brettanomyces* without killing the more SO_2_-sensitive *Saccharomyces* wine strains. The AWRI offered a welcome rendezvous for the ancient art of winemaking and the cutting-edge science of contemporary yeast biology.

##### A fresh smell of passionfruit in wine

Our flavour-active yeast research program focused on understanding how the metabolism of wine yeast interacts with grape juice in the wine production environment, and how this affects wine sensory properties (Swiegers et al. 2007). Practical outcomes from our team provided winemakers with tools to modulate aroma and flavour. Examples included novel yeast strains that produce less H_2_S during fermentation, and yeast blends that modulate the balance of esters and volatile thiols in Sauvignon Blanc.

Building on earlier work, we developed a prototype wine yeast able to enhance the passionfruit aroma in white and red wines. Our team integrated a gene cassette containing a bacterial carbon-sulfur lyase gene into the genome of a wine yeast. This resulted in the release of elevated levels of the volatile thiols 4-mercapto-4-methylpentan-2-one (4MMP) and 3-mercaptohexanol (3MH) from the corresponding cysteine conjugate precursors. The work showed it is possible to enhance significantly the aroma of wine, even from grapes where the flavour precursors are limited. This prototype (proof-of-concept) aroma-enhancing yeast was useful as a scientific benchmark model only; information generated with this prototype yeast was then applied to develop further non-GM yeast strains and inoculation strategies to enhance the varietal aromas of wines to predetermined market specifications, including an expression of regionality (Swiegers et al. 2007).

##### Uncorking raspberry wine with the first semisynthetic yeast

Another project focussed on raspberry ketone. This compound is the primary aroma compound found in raspberries, and naturally-derived raspberry ketone is a valuable flavouring agent. The economic incentives for the production of raspberry ketone, combined with the very poor yields from plant tissue, make this compound an excellent target for production by synthetically-engineered microbial strains. The AWRI team assembled a *de novo* pathway for the production of raspberry ketone using four genes from other species, encoding phenylalanine/tyrosine ammonia lyase, cinnimate-4-hydroxlase, coumarate-CoA ligase 2, and benzalacetone synthase, in an industrial strain of *S. cerevisiae*. Synthetic protein fusions were also explored to increase yields of the final product. The final semisynthetic strain produced significant amounts of raspberry ketone in wine. This paved the way for further pathway optimization to provide an economic alternative to raspberry ketone derived from plant sources (Lee et al. 2016). The raspberry-ketone yeast is the world’s first example of a semisynthetic wine yeast strain.

The above examples are only a few breakthroughs achieved by the AWRI’s yeast team. I am very proud of the AWRI’s achievements, and collaboration with my former colleagues there is ongoing. For example, we worked together to construct a synthetic *S. cerevisiae* pan-genome neo-chromosome (Kutyna et al. 2022). Our goal was to address the fact that laboratory yeast strains lack many of the genes that provide phenotypic diversity to industrial and environmental isolates. We designed and constructed a synthetic neo-chromosome that contains many of these diverse pan-genomic elements.

##### So good but time to move on

By the end of my tenure, Team AWRI had tripled its external research income, significantly expanded its global footprint in yeast biotechnology, and moved into a brand-new purpose-built and well-equipped facility, the Wine Innovation Cluster located on the Waite campus of Adelaide University. While I was the Managing Director of the AWRI, I also held an Adjunct Professorship at Adelaide University and an Honorary Professorship at the University of British Columbia (Vancouver, Canda). These were some of my best years in wine science. After nine years at the helm of the AWRI, it gave me great comfort to hand over the AWRI reins to mentee, Dan Johnson.

#### Expanding into university leadership and synthetic yeast genomics

##### Building a biofoundry capability

**Figure 10:**
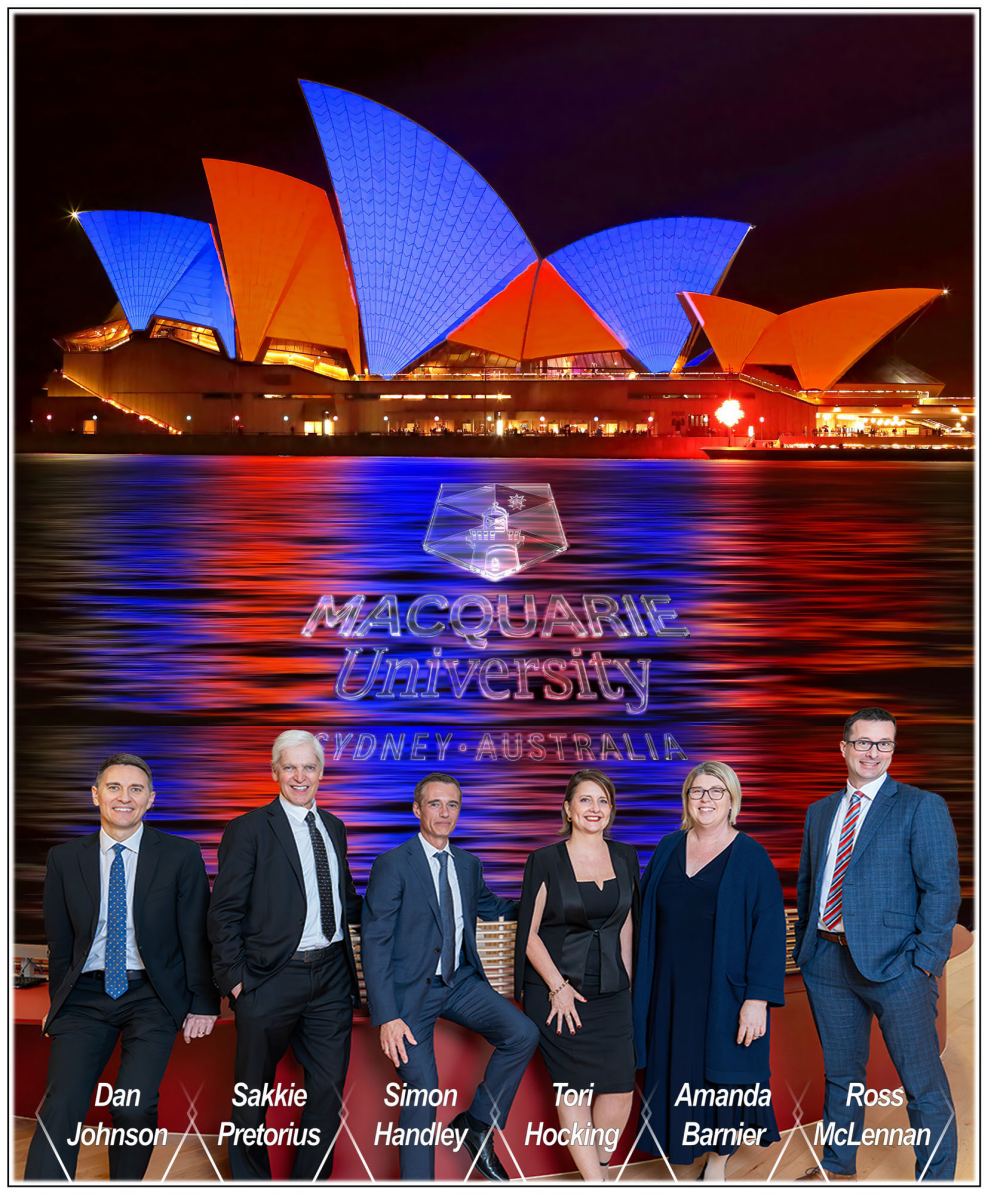
Leading Macquarie University’s research strategy as Deputy Vice-Chancellor (Research) since 2013. Founded in 1964 in Sydney as Australia’s entrepreneurial university, Macquarie University is set on a park-like green campus in the heart of Australia’s largest and fastest-growing business and technology precinct, *Macquarie Park Innovation District*. Sakkie is supported by an outstanding DVCR leadership team (left to right): Professor Dan Johnson (Pro Vice-Chancellor: Research, Innovation & Enterprise); Professor Simon Handley (Pro Vice-Chancellor: Graduate Research); Ms Tori Hocking (Executive Director: Research); Professor Amanda Barnier (Pro Vice-Chancellor: Research Performance & Development); and Dr Ross McLennan (Pro Vice-Chancellor: Research Services).

**Figure 11:**
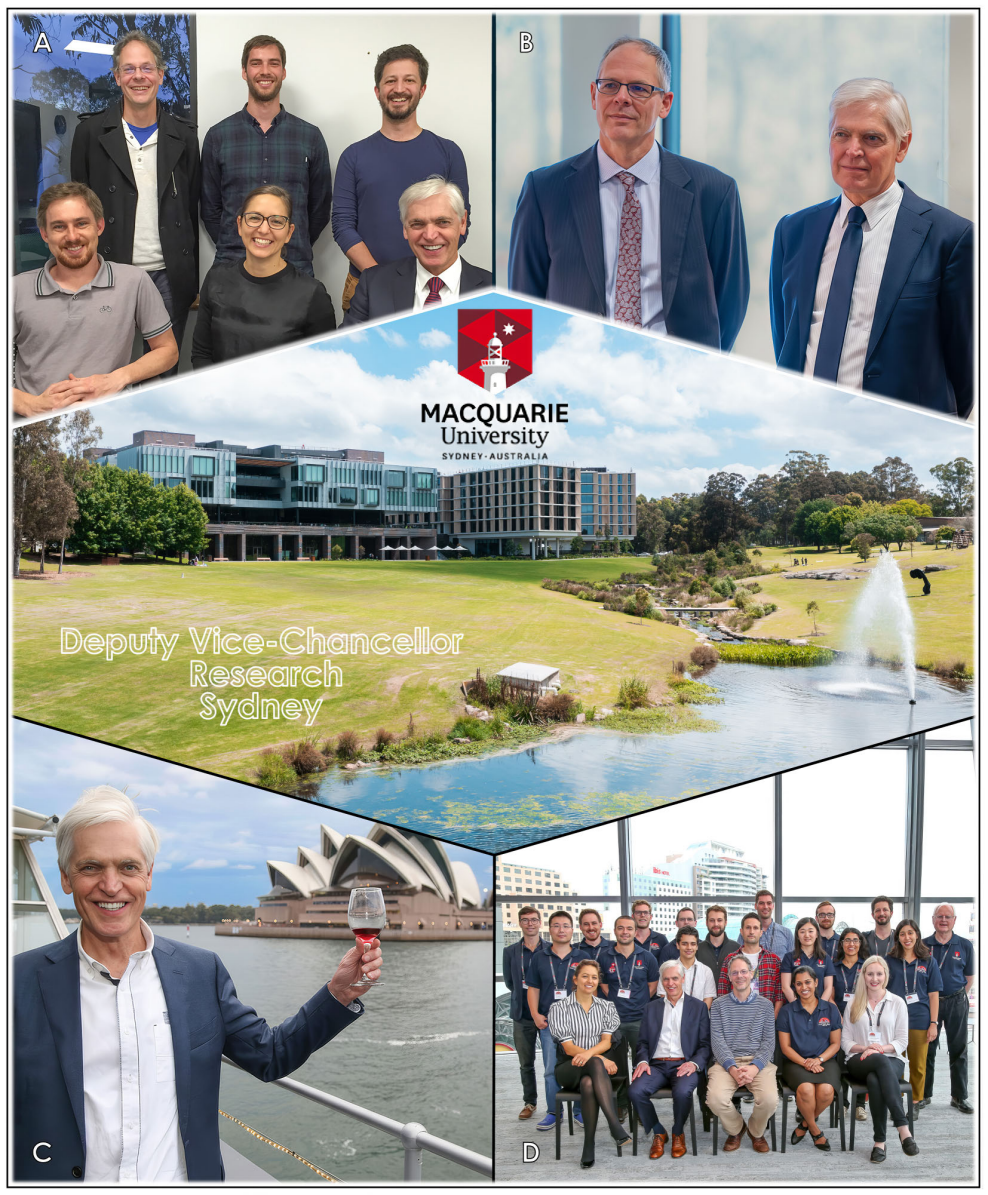
Co-leading Macquarie University’s synthetic biology initiative with Professor Ian Paulsen. (A) Dr Heinrich Kroukamp, Dr Natalie Curach, and Professor Sakkie Pretorius in the front row, and Professor Ian Paulsen, Dr Tom Williams, and Dr Hugh Goold in the back row. (B) Professor Ian Paulsen and Professor Sakkie Pretorius. (C) Sakkie celebrating Macquarie’s Synbio achievements on Sydney Harbour. (D) Macquarie’s *Yeast 2.0* team.

In 2011, I moved into higher education leadership, first as Deputy Vice-Chancellor (Research & Innovation) at the University of South Australia in Adelaide, and then, in 2013, into my current role as Deputy Vice-Chancellor (Research) at Macquarie University in Sydney (Fig. 10). While this shift took me further from the bench, it opened new possibilities to shape innovative research ecosystems at scale. At Macquarie, together with Ian Paulsen and a whole raft of enthusiastic postdoctoral research fellows and graduate research students, we established Australia’s first synthetic yeast genomics laboratory and biofoundry—the Australian Genome Foundry—and the Australian Research Council’s *Centre of Excellence in Synthetic Biology* (Fig. 11).

##### Inspired by nature and designed by science

A key project for our Synbio team here at Macquarie is our participation in the international *Synthetic Yeast Genome* project (known as the *Yeast 2.0* or *Sc2.0* project) led by Professor Jef Boeke from New York University (see Pretorius and Boeke 2018 and references therein). Being part of this ambitious project is one of the most challenging, but exciting, scientific adventures of my career. Sc2.0 emerged as a global collaborative effort to redesign and synthesize the entire *S. cerevisiae* genome from scratch. It presented a unique set of opportunities and challenges that prompted the use of rational design principles to enhance genome stability, flexibility, and engineering potential. Key features and changes to the native genome included the removal of introns, transposable elements and repetitive DNA; introducing loxPsym sites for genome rearrangement (via the SCRaMbLE system); stop codon reassignment; and an efficient design to support future genome minimization and reprogramming. Each synthetic chromosome followed the same design principles used in the assembly of the first semi-synthetic chromosome, *synIXR*, and was subsequently constructed by different international teams. Our team at Macquarie successfully synthesized and assembled chromosomes XIV and XVI: two of the sixteen man-made chromosomes which will soon constitute the world’s first synthetic eukaryote genome (see Erpf et al. 2025 and references therein).

##### Eleven labs arrive at the same place

Some of the most valuable lessons from the Sc2.0 project arose from the identification of ‘bugs’ following integration of synthetic DNA (Erpf et al. 2025). Eleven laboratories constructed one or more designated chromosome(s) following standardized consortium design principles. Despite the methodological differences, a striking pattern emerged: many of the same sequence-level issues and assembly ‘bugs’ appeared across the consortium. Recurring errors were traced to two main sources: design decisions that later proved problematic; and spontaneous, complicated mutations that arose during construction. However, by working together over the past 15 years, we solved these issues and now have 16 functional synthetic yeast chromosomes, ready to be consolidated in a single cell.

##### Heart of the frontier—people

**Figure 12:**
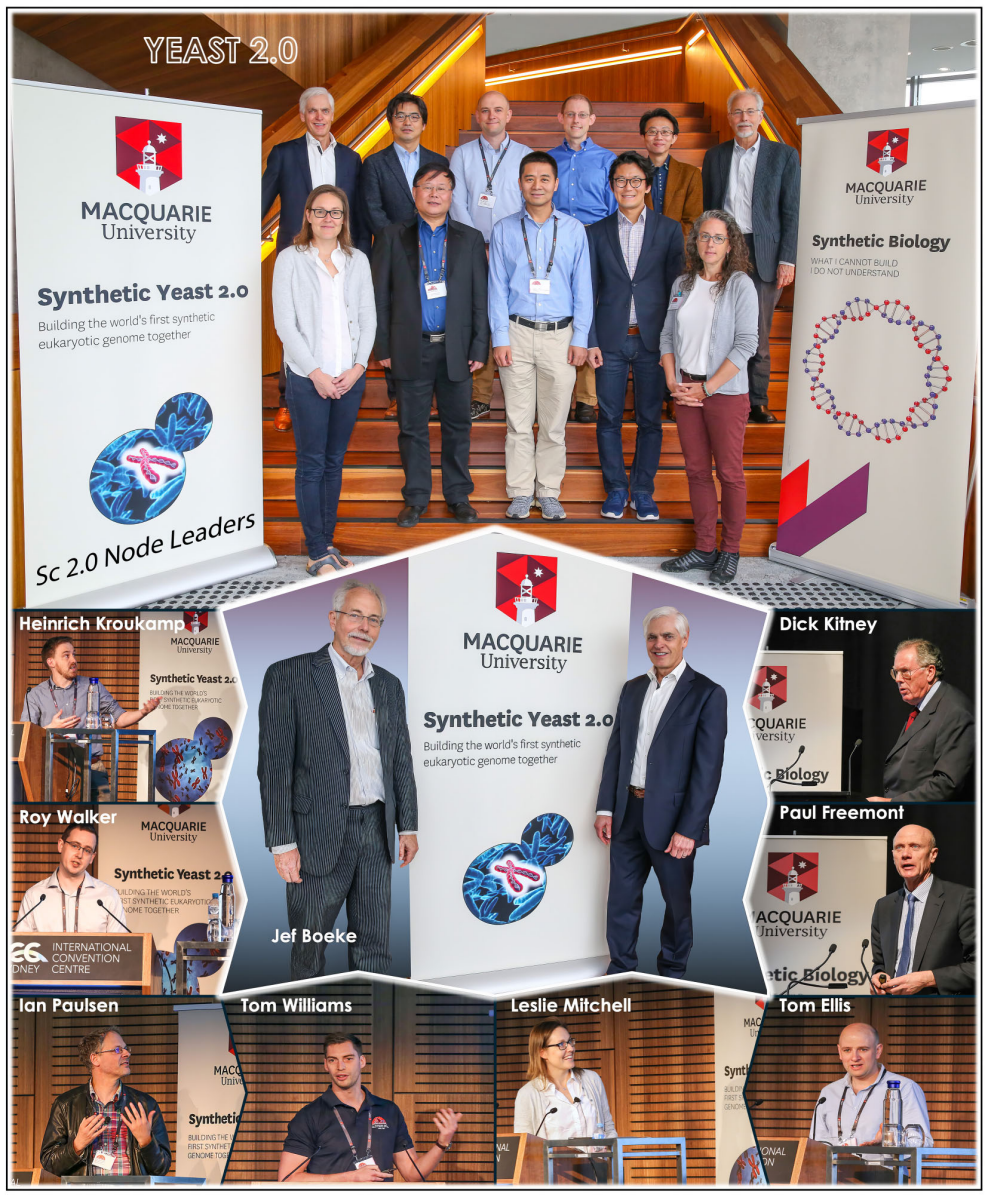
Hosting the *7th International Yeast 2.0 and Synthetic Genomes Conference* in Sydney in 2018. The Sc2.0 Node leaders (left to right) in the front row are Leslie Mitchell (USA), Yingjin Yuan (China), Junbiao Dai (China), Matthew Chang (Singapore), and Debra Mathews (USA); and in the back row, Sakkie Pretorius (Australia), Yasunori Aizawa (Japan), Tom Ellis (UK), Joel Bader (USA), Patrick Cai (UK), and Jef Boeke (USA).

Hosting the *7^th^ International Yeast 2.0 and Synthetic Genomes Conference* in 2018 in Sydney, and working with leading scientists across Australia, USA, UK, China, Japan and Singapore reminded me that yeast science remains at the heart of frontier discovery and innovation (Fig. 12).

Beyond the scientific milestones, what stands out most to me are the people—the students and postdocs whose energy and curiosity sustained my research; the collaborators who became lifelong friends; and the yeast community, which has been like an extended family. Serving on international editorial boards of peer-reviewed journals, international commissions, and conference committees has given me a deep appreciation for the collegial spirit that defines our field in yeast biology.

## From hindsight to insight and foresight

### Lessons carried forward from a lifetime in science

Looking back over nearly five decades in yeast research, one of the most striking changes I have witnessed is the accelerating pace of discovery. The scientific world I entered as a young student bears little resemblance to the one we inhabit today. Yet amid extraordinary technological and societal change, one constant has endured: human curiosity—the impulse to ask questions, to test ideas, and to push beyond what is known.

My own career has unfolded at the intersection of design and serendipity. Some advances emerged from carefully planned experiments; others arose unexpectedly, revealing new directions only in hindsight. Together, these experiences reinforced a lesson that has guided my work: progress in science is rarely linear, but it is cumulative. Each insight, whether incremental or transformative, builds on what came before and subtly reshapes what becomes possible next.

### Learning to navigate change rather than resist it

Experience has taught me that time moves like a constantly flowing stream. We cannot halt its course or step outside its movement, but we can learn how to navigate its shifting currents. This metaphor feels particularly apt in periods of uncertainty, when the future seems poised to diverge sharply from the past.

Throughout my career—from early molecular yeast genetics to synthetic genomics—I have seen entire fields transformed by new tools and ideas. The most productive response has never been to resist change, nor to be swept along uncritically, but to remain attentive: to understand emerging directions early, to ask how they connect to established knowledge, and to adapt research questions accordingly. Retrospection, in this sense, is not backward-looking nostalgia, but a discipline for making sense of change.

### Converging technologies through a yeast biologist’s lens

In recent years, multiple technological streams have converged with unusual force. Advances in synthetic biology, computation, automation, and materials science are reshaping how biological research is conducted and applied (see Pretorius et al. 2025 and references therein). From my vantage point as a yeast biologist, these convergences are not abstract trends but lived realities—encountered in genome synthesis, biofoundries, and increasingly data-driven experimentation.

What distinguishes the present moment from earlier periods of innovation is not simply the power of individual technologies, but their interaction. Biology has become an engineering substrate; computation has become a biological design partner. These shifts have challenged long-held assumptions about how we generate knowledge, train scientists, and translate discovery into impact.

### Yeast as a bridge between biology and engineering

Yeast has repeatedly proven to be an ideal organism for navigating these transitions. Its evolutionary history has optimized it to manage information and energy efficiently, while its experimental tractability has made it a natural platform for synthetic genomics. Advances in DNA synthesis, genome design, and computational modelling have allowed us to move from reading yeast genomes to rewriting them.

The completion of 16 chemically synthesized chromosomes in *S. cerevisiae* naturally invites speculation about what might come next (see Pretorius et al. 2025 and references therein). Rather than offering predictions, I see this as an invitation to reflect on how far the field has travelled—and how foundational research, sustained over decades, makes such questions possible in the first place.

### Insight before foresight

**Figure 13:**
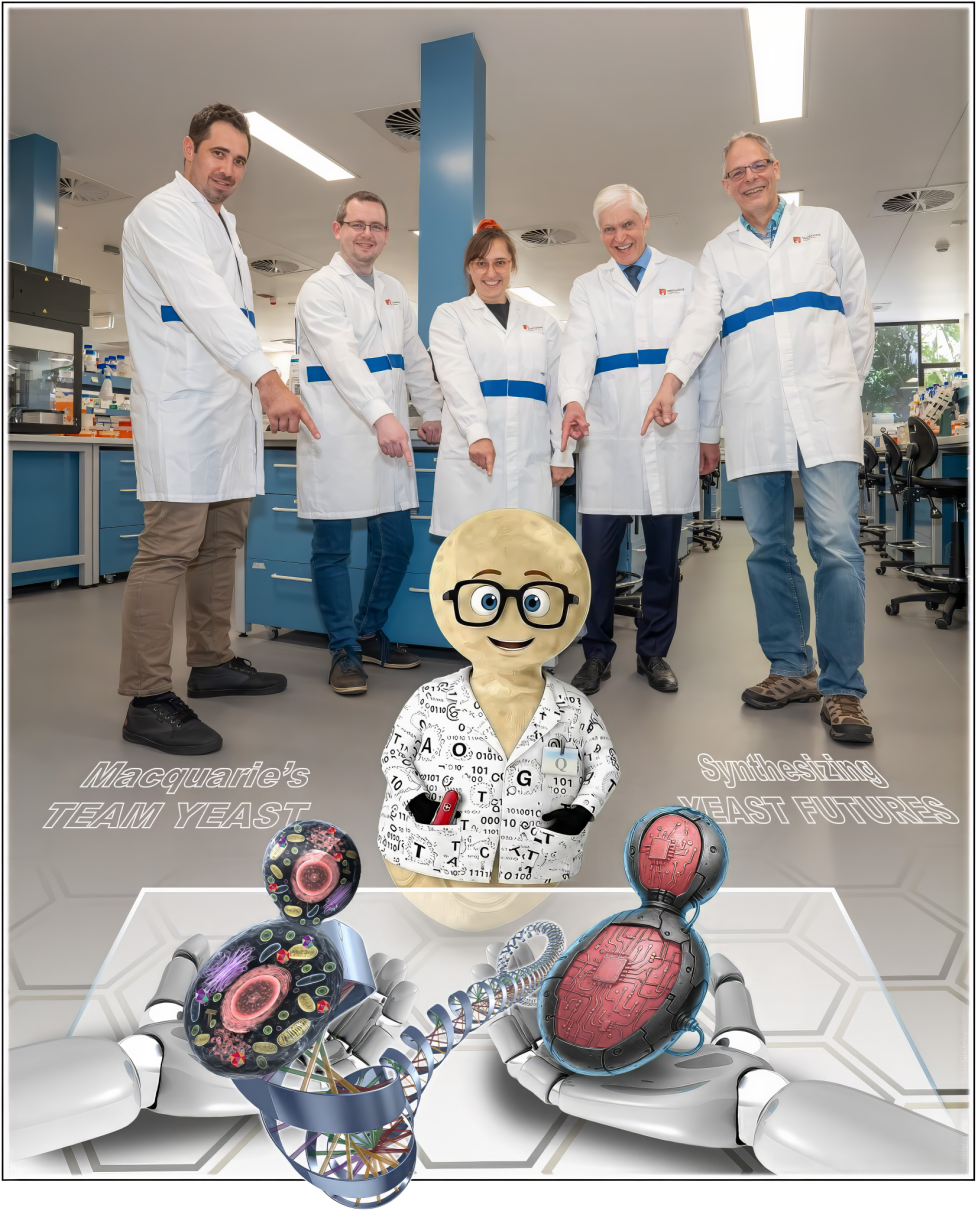
Macquarie University’s Team Yeast aiming to integrate artificial intelligence, semiconductor nanochip technology, and engineering into synthetic biology. From left to right: Dr Edward Archer, Dr Roy Walker, Dr Paige Erpf, Professor Sakkie Pretorius, and Professor Ian Paulsen.

One lesson that retrospection makes clear is that foresight without insight is fragile. Throughout my career, the most durable advances have been those grounded in deep biological understanding, tested cautiously, and guided by ethical and societal awareness. As biology increasingly interfaces with computation, electronics, automation, and engineering this principle becomes more—not less—important. A couple of years ago, our Synbio team here at Macquarie embarked on the integration of artificial intelligence, synthetic biology and nano-engineering (Fig. 13). So far, the learnings have been profound and the possibilities infinite.

History reminds us that transformative technologies are neither inherently beneficial nor inherently dangerous. Their impact depends on the values, intentions, and governance frameworks that shape their use. Sound ethical, regulatory, and societal considerations—including political, economic, social, technological, environmental, and legal dimensions—have repeatedly proven essential in translating innovation into lasting benefit.

### From reflection to responsibility

For me, the purpose of looking back is not to predict the future, but to clarify responsibilities in the present. The convergence of biological and informational technologies offers extraordinary opportunities for discovery and application, but also calls for humility, restraint, and collaboration across disciplines.

If retrospection teaches anything, it is that progress is most enduring when curiosity is paired with care, ambition with perspective, and innovation with responsibility. Yeast has been my guide through many such transitions, and it continues to remind me that even the most sophisticated technologies ultimately rest on simple biological principles—patiently uncovered, collectively shaped, and thoughtfully applied.

## From specifics to broader reflections

### Learning from the past to lead the present and secure the future

My mantra has always been to genuinely *respect the past*, to wisely *lead the present*, and to do our utmost to *secure a better future* for all. However, the blistering pace of today’s converging technologies means that while hindsight might be 20/20, foresight is 50/50. Therefore, we must keep an open mind in everything we consider and do. In the technology-driven *hype-horror-hope* era in which we find ourselves today, we should not be tempted to either glorify these general-purpose transformative technologies as a panacea to all ills or to crucify them as an unavoidable danger. We should always seek clarity before we critique or criticize.

Technology *per se* is not evil; it is a *tool*. General-purpose technologies: the *Technology of Intelligence* (artificial general intelligence) and the *Technology of Life* (synthetic biology)—augmented by other transformative technologies, such as quantum, robotics, and nano-engineering—belong in this category. A hammer is a tool: it can be used to build a house or smash a window. The issue is not the tool; it’s the mindset and mind behind the hand that holds it.

We are living at a pivotal turning point in the *Age of Technovation*, unfolding alongside the erosion of the post-1945 world order. These general-purpose, transformative technologies are no longer distant prospects; they are already reshaping how we work, how we learn, and how we connect. Yet at their core, they remain just that—tools. Like the wheel, controlled fire, the printing press, the combustion engine, electricity, and the internet, technologies such as artificial general intelligence and synthetic biology possess no moral compass of their own. It is the mindset behind the hand that wields them that determines whether they bring shared prosperity or profound harm.

### A garden of opportunity

Retrospectively, I can see that history offers us stories that remind us of this truth in science and innovation. Metaphorically speaking, when the *Garden of Eden* became the *Garden of Exile*, forcing people to move eastward, humanity first learned how to bake bricks in the ancient city of Babylon. It was a breakthrough. Bricks gave them the power to build durable homes, stronger cities, and new possibilities for human community. It was a moment of genuine innovation in the cradle of civilization. But soon, the motivation shifted. With those very same bricks, people decided to build a tower that would reach the heavens—not to improve life, but to make a name for themselves. What began as a tool for shelter became a monument of pride and arrogance—a wall dividing communities.

Like in ancient times and during my research career, I can see that same tension is still alive in our age of integrating transformative technologies. The lessons I learned over my career taught me that these tools can be used to understand the intricacies of yeast cells, to cure disease, and to unlock creativity in tackling humanity’s hardest problems—or they can be used to harm, to divide, to exploit, and to pursue power without accountability in a deglobalizing world.

The lesson is clear: it is not the tool that determines the outcome—it is us. Our intentions, our values, our choices will shape whether this moment becomes our *Garden of Opportunity* or our *Tower of Confusion* and *Wall of Division*. So, the challenge before us is to step into the future not with fear, and not with arrogance, but with imagination and humility. Let us harness yesterday’s learnings and today’s transformative technologies not to make a name for ourselves, but to make a positive difference for others. Because in the end, technology will not decide our story. We will. As a wine scientist turned synthetic yeast biologist, I see this vividly.

### Reflecting forward

**Figure 14:**
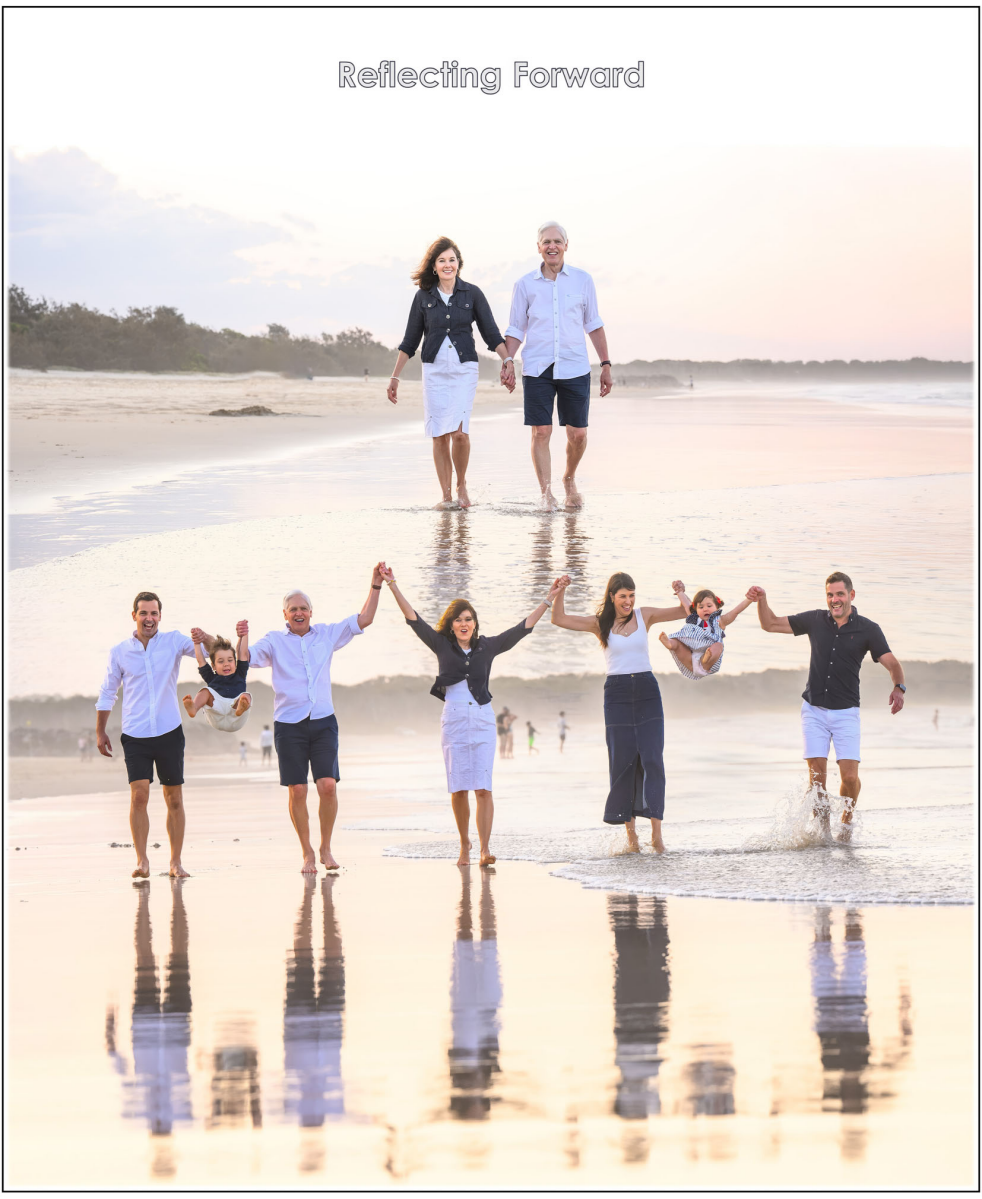
Grateful hearts reflecting forward with the deeply-connected Pretorius family. From left to right: Pappa Stefan, Prince Louis, Oupa Sakkie, Ouma Liesie, Mamma Stephanie, Princess Gigi, and Uncle Bernie. Fourteen generations on, this is a Pretorius family—perfect in its imperfections. It is a family bound together by unbreakable bonds. This family is my lifeblood and the air that I breathe.

As I reflect on my life journey, I feel immense gratitude to my grandparents and my parents; my teachers, mentors, students and colleagues; and above all, my wife Elize, our sons Bernard and Stefan, my daughter-in-law Stephanie, and our grandchildren, Louis and Giorgia—the fourteenth generation of Pretoriuses born in Australia—for making it all possible. They inspire me. They give me hope. They raise me up to more than I can be. My family is my lifeblood and the air that I breathe (Fig. 14), and discovery is the fire in my belly. Yeast has been both my teacher and my companion, and it continues to astonish me with its capacity to reveal life’s secrets and to power new technologies. If there is one lesson I would pass on, it is this: never underestimate the potential of small beginnings—whether a microscopic yeast cell or a fledgling idea, or a humble boy from a subsistence farm—to spark discoveries that can change the world.
